# Enhancing the outcomes of bariatric surgery with inhibitory control training, electrical brain stimulation and psychosocial aftercare: a pilot study protocol

**DOI:** 10.1186/s40337-024-01160-3

**Published:** 2024-12-09

**Authors:** Sarah A. Rösch, Lennart Wünsche, Carsten Thiele, Therese Reinstaller, Tino Zähle, Kathrin Schag, Katrin E. Giel, Christian Plewnia, Johann Steiner, Florian Junne

**Affiliations:** 1 University Clinic of Psychosomatic Medicine and Psychotherapy, University Hospital, Otto von Guericke University Magdeburg, Leipziger Str. 44, 39120 Magdeburg, Germany; 2 German Center for Mental Health (DZPG), Partner Site Halle-Jena-Magdeburg, Magdeburg, Germany; 3 Center for Intervention and Research on Adaptive and Maladaptive Brain Circuits Underlying Mental Health (C-I-R-C), Halle-Jena-Magdeburg, Magdeburg, Germany; 4 Department of Neurology, University Hospital, Otto von Guericke University Magdeburg, Magdeburg, Germany; 5 Center for Behavioral Brain Sciences (CBBS), Magdeburg, Germany; 6 Visceral, Vascular and Transplant Surgery, University Clinic for General, University Hospital, Otto von Guericke University Magdeburg, Magdeburg, Germany; 7 Department of Psychosomatic Medicine und Psychotherapy, Medical University Hospital Tübingen, Eberhard Karl University Tübingen, Tübingen, Germany; 8 Center of Excellence for Eating Disorders, Tübingen, Germany; 9 Department of Psychiatry and Psychotherapy, Medical University Hospital Tübingen, Eberhard Karl University Tübingen, Tübingen, Germany; 10 Department of Psychiatry, University Hospital, Otto von Guericke University, Magdeburg, Germany; 11 Laboratory of Translational Psychiatry, University Hospital, Otto von Guericke University Otto-von-Guericke-University Magdeburg, Magdeburg, Germany; 12 Center for Health and Medical Prevention (CHaMP), Magdeburg, Germany

**Keywords:** tDCS, Bariatric surgery, Inhibitory control training, Food-specific impulsivity, Psychosocial intervention, Aftercare, Obesity surgery

## Abstract

**Background:**

Notwithstanding the documented short- and long-term weight loss and remission of physical and mental diseases following bariatric surgery, a significant proportion of patients fail to respond (fully) to treatment in terms of physical and mental health improvement. Mounting evidence links food-specific impulsivity, prefrontal cortex (PFC) hypoactivity and disrupted hormone secretion in bariatric surgery candidates to poorer post-surgical health outcomes. Neuromodulatory treatments like transcranial direct current stimulation (tDCS) uniquely target these neurobehavioral impairments. We present a pilot study protocol offering tDCS combined with an inhibitory control training and a structured psychosocial intervention to patients after bariatric surgery.

**Methods:**

A total of *N* = 20 patients are randomized to 6 sessions of verum or sham tDCS over the PFC, combined with an individualized food-specific inhibitory control training and a structured psychosocial intervention within 18 months after bariatric surgery (t0). Beyond acceptability, feasibility and satisfaction of the intervention, effects of verum versus sham tDCS on food-specific impulsivity and on secondary outcomes quality of life, general impulsivity and psychopathology, food-related cravings, eating disorder psychopathology, weight trajectory and endocrine markers are assessed 4 weeks (t1) and 3 months after the intervention (t2).

**Discussion:**

Results will provide information on the potential of combining tDCS with an inhibitory control training and a structured psychosocial intervention to enhance physical and mental outcomes after bariatric surgery. The present study may guide the development of future research with regard to tDCS as a brain-based intervention and of future post-surgical clinical programs, paving the way for randomized-controlled trials in larger samples.

**Trial registration:**

The trial was prospectively registered on July 8, 2024, under the registration number DRKS00034620 in the German Clinical Trials Register (https://drks.de/search/de/trial/DRKS00034620).

**Supplementary Information:**

The online version contains supplementary material available at 10.1186/s40337-024-01160-3.

## Background

The prevalence of obesity, defined by a body-mass-index (BMI) ≥ 30 kg/m² [1], has reached pandemic levels, affecting 19.0% of the German population [2]. Obesity carries a substantial risk for chronification and increases the odds for secondary diseases [3], challenging public health and economic systems [4]. Bariatric surgery, recommended for severe obesity (BMI ≥ 40 kg/m²) or lower-graded obesity with comorbidities or failed conservative treatment [5, 6], effectively reduces weight and improves obesity-related physical and mental health. Remaining challenges lie in inadequate weight loss, weight stabilization, or weight regain after an initial weight nadir [7], which are associated with higher anxiety and depressive symptoms and dysfunctional eating patterns [8]. Access to structured postoperative interventions remains limited, notwithstanding their clinical recommendation [5] and beneficial influence on functional eating behaviors and psychosocial well-being [9–11].

State impulsivity^1^ and food-specific impulsivity^2^ as prominent features of bariatric surgery candidates are not addressed in the regular pre- and postsurgical procedures, despite their presumed negative impact on outcomes [12–15]. Impulsivity supposedly promotes dysfunctional eating behaviors [16–18], which undermine bariatric surgery outcomes [19–21]. Compelling evidence shows a possibly causal bidirectional link between food-specific impulsivity and reduced prefrontal cortex (PFC) activity in obesity [22], with variations in PFC functionality explaining differences in dietary self-regulation, while weight aberrances impair PFC functionality.

Building on these neurobiological peculiarities, neuromodulatory treatments enjoy increasing popularity for eating and weight disorders [23, 24]. Transcranial direct current stimulation (tDCS), a non-invasive, safe, and tolerable treatment targeting the neuronal excitability of certain brain regions through a weak direct current emitted via electrodes on the head [24, 25], showed promise for chronic pain, mental disorder [26], and recently for binge-eating disorder (BED) treatment [27, 28]. Beyond feasibility, safety and acceptability of tDCS combined with an inhibitory control training, reductions in binge-eating episodes and improvements in secondary outcomes (e.g., BMI) were shown at post-treatment and 3-month follow-up in a prospective randomized-conrolled pilot study comprising *n* = 41 patients with BED. Importantly, active versus sham tDCS was associated with significantly less binge-eating episodes at follow-up [27, 28].

Recent attention has additionally focused on the interaction between postoperative hormonal changes and neural processes, particularly in modulating caloric intake post-surgery [29–32]. Variations in the secretion of ghrelin, peptide YY (PYY), and glucagon-like peptide-1 (GLP-1) differentiated individuals who responded well to bariatric surgery regarding weight loss from those who did not in fasting and postprandial states [29]. This variability in post-surgical hormonal response may stem from the brain’s secretion and production of these hormones, influencing food-specific processing and hedonic eating patterns [30].

### Purpose

The aims of this pilot study are to assess the feasibility, acceptability and additive effects of tDCS combined with an inhibitory control training and a structured intervention on diverse outcomes after bariatric surgery. Prefrontal hypoactivity is targeted through tDCS to reduce food-specific impulsivity and dysfunctional eating behaviors, while a structured intervention supports lifestyle modifications (e.g., nutrition, exercise).

## Materials and methods

### Sample and recruitment

A total of *N* = 20 pre- or post-surgical patients are recruited from the University Clinic for General, Visceral, Vascular and Transplantation Surgery Magdeburg through flyers and verbal information. Patients receive written information on the study and provide written informed consent. Table 1 depicts inclusion and exclusion criteria.

**Table 1:** Inclusion and exclusion criteria

Inclusion criteria	Exclusion criteria
Age ≥ 18 years	Insufficient knowledge of German language
BMI ≥ 35 kg/m²	Current serious mental or neurological disorders
Sleeve gastrectomy in the past 18 months or planned sleeve gastrectomy	Current intake of tranquilizers or neuroleptics
Written informed consent	Current intake of GLP-1-receptor agonists
	Current intake of dopaminergic medication
	Previous neurosurgical procedures or traumatic brain injury
	Previous alcohol or drug abuse
	Current pregnancy or lactation
	Insufficiently adjusted Diabetes mellitus Type 2
	Metal or electric implant in the head, e.g. cochlea implants
	Pacemakers

Abbreviations. BMI, body-mass-index; GLP-1, glucagon like peptide-1

### Sample size

Due to the lack of studies on tDCS after bariatric surgery, the expected group difference remains unknown. Although a sample size calculation was not conducted (see also the precursor study in BED; 27, 28), the sample size seemed adequate for feasibility studies [33].

### Randomization and blinding

Patients are pseudo-randomly assigned to verum or sham tDCS in a 1:1 ratio. Using the stimulation device’s ‘study mode’ for operator-blinded device activation, an a-priori created 5-digit code from a computer-generated list is entered, automatically applying the correct condition without input from the therapist and thus ensuring double-blindness. Typically, tDCS causes sensations (itching, tingling), which quickly wane due to somatosensory adaptation processes [34, 35]. Patients in sham tDCS receive 43 s stimulation at the beginning of each trial to mimic these sensations. After the final stimulation session, patients guess their assigned condition and indicate their level of confidence in their guess.

### Assessment protocol

The study comprises a baseline assessment before tDCS (t0), an assessment before and after each tDCS session, a post-assessment 4 weeks after tDCS with the inhibitory control training and the initial psychosocial group sessions (t1) and a follow-up assessment 3 months after the intervention (t2). During each assessment, patients are interviewed on eating and other mental disorders, fill in questionnaires and blood samples are taken before and after a standardized liquid meal (Table 2).

**Table 2:** Assessment points

Measure	t0	t1	t2
Sociodemographic data	x		
**Primary Outcomes**			
Feasibility and acceptance		x	
Food-specific impulsivity	x	x	x
**Secondary Outcomes**			
Quality of life	x	x	x
Eating disorder psychopathology	x	x	x
General impulsivity	x	x	x
Food-related cravings	x	x	x
General psychopathology	x	x	x
Mental disorder comorbidity	x	x	x
Weight	x	x	x
Secretion of ghrelin, PYY and GLP-1	x	x	x

Abbreviations. GLP-1, glucagon like peptide-1; PYY, peptide YY; t0, baseline after the sleeve gastrectomy; t1, 4 weeks after the tDCS intervention; t2, 3 months after the tDCS intervention

Note. Session-wise assessments are described in-text

### Primary outcomes

#### Feasibility and acceptance

Feasibility and acceptance are estimated through the percentage of included patients from eligible patients at t0, the drop-out rate through t0 to t2 (i.e., the 3-month follow-up) and a self-developed questionnaire on patients’ need and motivation for training uptake, feasibility, acceptability and overall satisfaction with the treatment.

#### Food-specific impulsivity

Food-specific impulsivity is assessed behaviorally through error rate and latency in the antisaccade task, through the self-report Three Factor Eating Questionnaire (FEV; 36, 37) measuring dietary practices via 51 items on *restraint of eating behavior*, *disinhibition of control* and *perceived hunger* and through an own translation of the Food Craving Acceptance and Action Questionnaire (FAAQ; 38; Supplementary Material) including 10 items on the *ability to regulate eating despite urges and cravings* and on the *desire to maintain internal control over eating thoughts*.

### Secondary outcomes

#### Food-related cravings

The Food Cravings Questionnaire-Trait-reduced FCQ-T-r [39, 40] measures food-related cravings with 15 items.

### Quality of life

The WHO-5 well-being index [41] comprises 5 items measuring subjective well-being over the last 2 weeks.

### Eating disorder psychopathology

Eating disorder psychopathology is measured through the Eating Disorder Examination Interview – Bariatric Surgery Version (EDE-BSV; 42), providing information about *restraint*, *eating concern*, *weight concern*, and *shape concern* over the past 28 days and on surgery-specific *plugging* and *dumping*. Importantly, the EDE-BSV differentiates inappropriate compensatory behaviors aimed at preventing weight gain (e.g., excessive exercise) for weight and shape reasons from behaviors occuring as a consequence of bariatric surgery. The self-report Eating Disorder Examination Questionnaire (EDE-Q; 43–45) includes six diagnostic items and 22 items depicting *restraint*, *eating concern*, *weight concern*, and *shape concern* over the past 28 days. Lastly, the Quality of Life for Obesity Surgery (QOLOS; 46) self-report questionnaire assesses health-related quality of life with 36 pre-and postoperative items measuring *eating disturbances*, *physical functioning*, *body satisfaction*, *family support*, *social discrimination*, *positive activities* and *partnership*, and 20 post-operative items only depicting *excess skin*, *eating adjustment*, *dumping* and *satisfaction with surgery*.

### General impulsivity

General impulsivity is assessed with the short version of the Barratt Impulsiveness Scale (BIS-15; 47, 48), measuring *non-planning impulsivity*,* motor impulsivity* and *attentional impulsivity* with 15 items. The Impulsive Behavior-Scale (UPPS; 49) assesses *negative urgency*, *lack of premeditation*, *positive urgency* and *lack of perseverance* with 40 items. Common impulsive behaviors within the last seven days are measured with a self-developed protocol [27].

### Mental disorder comorbidity

Mental disorder comorbidity is assessed with the Mini-DIPS (Diagnostisches Kurz-Interview bei psychischen Störungen; 50), a structured diagnostic interview on the most common disorders based on the Diagnostic and Statistical Manual of Mental Disorders, Fifth Version [51] and the International Classification of Diseases, Tenth Revision [52].

### Depressive symptoms and general psychopathology

Depressive symptoms are rated using the Beck Depression Inventory (BDI II; 53) with 21 items. The Patient Health Questionnaire (PHQ-D; 54, 55) measures *depressive symptoms*, *somatic symptoms*, *anxiety symptoms*, *eating disorder symptoms* and *alcohol-related disorders* with 59 items.

### Endocrine markes

Patients are asked to attend the lab following a 10 h fasting period prior to blood sampling (i.e., at t0, t1, and t2; Table 2). The instruction to fast prior to the lab visit is given, orally during the screening procedure via telephone and in a written format via E-mail and via letter. To facilitate the fasting requirement, all diagnostic measurements are carried out between 8 and 12 am. After arriving at the lab, patients are asked whether they adhered to the fasting requirement. Possible violations of the fasting requirement are noted and accounted for in the statistical procedure. Peripheral blood samples are taken before and 30 min after a standardized liquid mixed meal (125 ml, 300 kcal; 12 g of protein, 12 g of fat, 37 g of carbohydrates, Nutricia Fortimel Compact, Nutricia Milupa GmbH, Hamburg, Germany). To analyze gut hormones, EDTA tubes are prefilled with aprotinin (500 KIU per ml blood) to avoid enzymatic hormone degradation. Tubes are cooled at 4 °C until centrifugation. After centrifugation at 4 °C for 15 min at a Relative Centrifugal Force of 1600 g, plasma is aliquoted into 0.5 ml Protein LoBind Tubes (Eppendorf) and stored at -80 °C. Measurements of GLP-1, PYY and total Ghrelin are performed using ELISA and EIA-Kits (Merck, Human PYY ELISA Kit:#RAB1078-1KT; GLP-1 EIA Kit:#RAB0201-1KT; Ghrelin EIA Kit:# RAB0207-1KT) in duplicates.

### Weight and height

Patients’ weight and height are assessed objectively on a calibrated scale to compute BMI (kg/m²).

### Session-wise assessments

Patients rate hunger and mood on a visual analogue scale, fill in the Food Craving Questionnaire-State (FCQ-S; 39, 40) before and after each session, and are asked about side effects after each session on a 5-point Likert scale from 1 = *not at all* to 5 = *very strong*. Other potentially severe adverse events are documented by the experimenter.

### Intervention

**Fig. 1:**
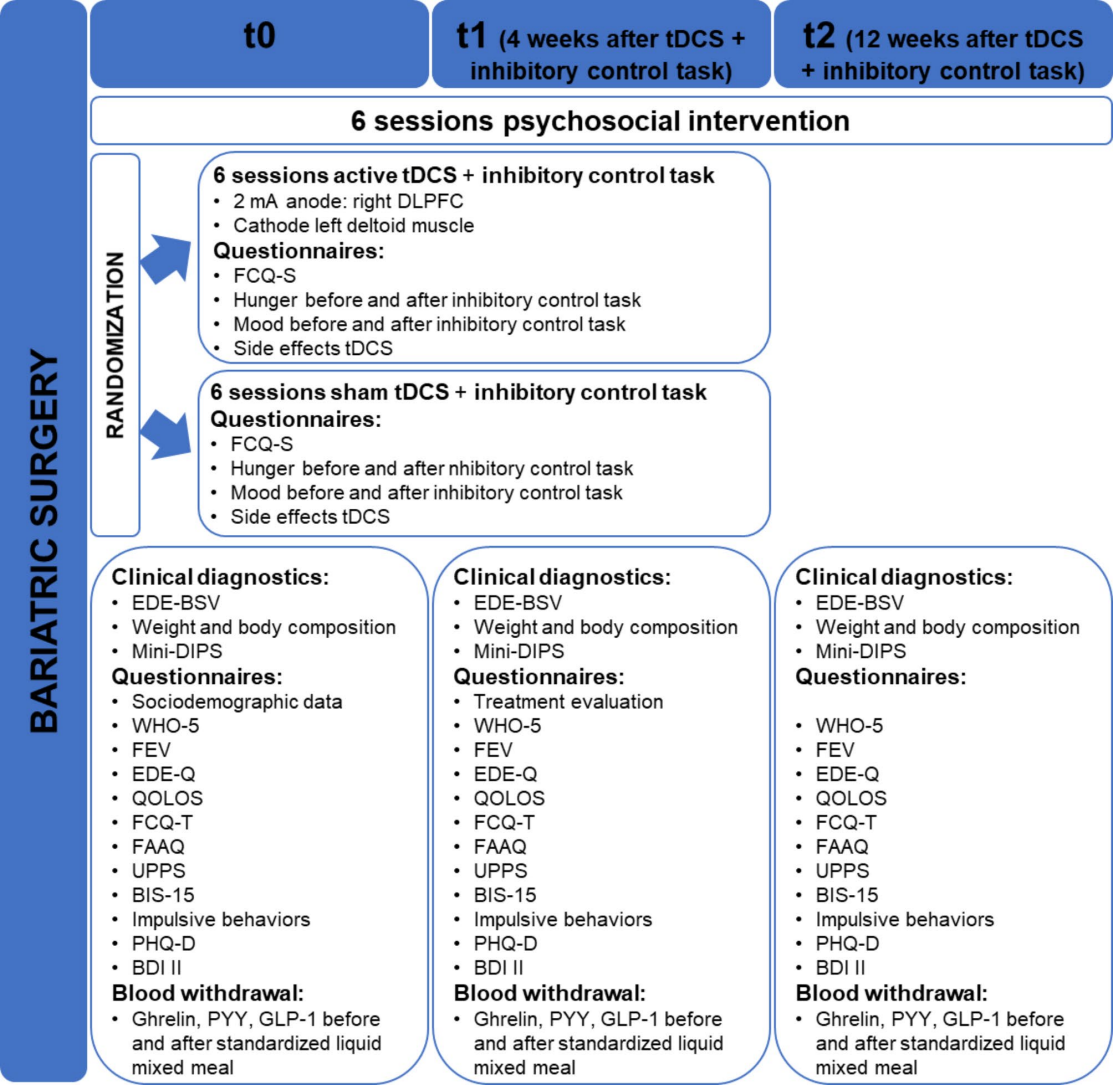
Study procedures and assessment points of the trial Abbreviations. BDI, Beck Depression Inventory; BIS-15, short version of the Barratt Impulsiveness Scale; DLPFC, dorsolateral prefrontal cortex; EDE-BSV, Eating Disorder Examination Interview – Bariatric Surgery Version; EDE-Q, Eating Disorder Examination Questionnaire; FAAQ, Food Craving Acceptance and Action Questionnaire; FCQ-S, Food Cravings Questionnaire-State; FCQ-T, Food Cravings Questionnaire-Trait-reduced; FEV, Three Factor Eating Questionnaire; GLP-1, glucagon like peptide-1; Mini-DIPS, Diagnostisches Kurz-Interview bei psychischen Störungen; PHQ-D, Patient Health Questionnaire; PYY, peptide YY; QOLOS, Quality of Life for Obesity Surgery; t0, baseline after the sleeve gastrectomy; t1, 4 weeks after the tDCS intervention; t2, 3 months after the tDCS intervention; tDCS, transcranial direct current stimulation; UPPS, Impulsive Behavior-Scale; WHO-5, well-being index

Figure 1 depicts the individual participant timeline.

### tDCS along with a food-specific inhibitory control training

Six sessions of a food-specific inhibitory control training with verum or sham tDCS over the right dorsolateral PFC [27, 28] are delivered twice weekly over 21 days. Patients initially receive a 5-10-min psychoeducation explaining the mechanisms of the training, the stimulation and the transfer of training effects to everyday life. At the first session, patients rate their liking and appetite of *n* = 40 pictures of high-caloric foods [56] on a 5-point Likert scale from 1 = *not at all* to 5 = *very much* or 1 = *very unappetizing* to 5 = *very appetizing*, respectively. The 20 pictures with the highest individual ratings are used for the training.

For tDCS, the electrodes are prepared with a Ten20 conductive paste (Weaver and Company, Aurora, CO, USA). A 5 × 7 cm rubber electrode serving as the anode is positioned at F4 in accordance with the international 10–20 system [57], with the shorter side of the electrode positioned horizontally. Another 5 × 7 cm electrode, serving as the cathode, is placed extracephalic on the left deltoid muscle. This unipolar montage is used to stimulate the right dorsolateral PFC. Electrodes are connected to a battery-driven stimulator (DC-Stimulator Plus, NeuroConn GmbH, Ilmenau, Germany). In the verum condition, the stimulator delivers 15 min of 2 mA direct current after an initial fade-in of 5 s, covering the antisaccade task time. Participants in the sham condition receive only 43 s of stimulation as a valid placebo condition prior to the start of the antisaccade task (see below).

During the inhibitory control training, participants’ gaze behavior is tracked using an Eyelink (Eyelink 1000, SR Research, Ottawa, Ontario, Canada) with a sampling rate of 500 Hz and gaze position accuracy of 0.25–0.50°. Each trial begins with a central fixation cross displayed for 1250 ms, followed by a 200 ms interstimulus interval and, only if the patient has correctly fixated on the fixation cross, an individual food picture presented slightly to the left or right of the cross in the peripheral visual field for 1000 ms. Patients are instructed to move their eyes in the opposite direction as quickly as possible after stimulus onset, i.e., to perform an antisaccade. An antisaccade directed away from the food picture is counted as correct, while a saccade toward the food picture is counted as an error. Each food item is presented four times per block with four blocks in total, with presentation locations counterbalanced between left and right, resulting in 320 total trials. Between each block, participants can take a self-timed pause up to 1 min. Participants receive feedback about the percentage of committed errors after each session.

### Group intervention

Patients undergo a monthly structured intervention based on cognitive-behavioral and psychodynamic principles and led by a psychologist and a qualified dietician, starting with the first tDCS session. Topics encompass lifestyle after surgery (including nutrition, physical activity, social relationships and support), dealing with vacation, weekends and public holidays, (problematic) eating patterns, body image, stress and emotion regulation and relapse prevention.

### Ethical aspects

Data is pseudonymized and entered manually by the study personnel. Questionnaires are given in paper versions and transferred to an electronic study database through a research assistant. Fidelity and plausibility is checked. All trial data is stored in line with the European General Data Protection Regulation 2018. The trial was approved by the Local Ethics Committee of the University Hospital of Magdeburg (reference number: 10/24). Patients provide written informed consent prior to participation and are allowed to withdraw from the trial at any point without disadvantage.

### Statistical methods

All statistical analyses are carried out using R version 4.0.2 [58]. Mixed models are used to assess primary and secondary outcomes between t0 through t2, with the time vs. treatment interactions at t1 and t2 as primary parameters. Reported *p*-values are interpreted non-confirmatorily against the small sample size. The primary analysis is conducted in the intent-to-treat population. Drop-outs are handled with an imputation model that uses 500 replications and includes age, sex and baseline assessment.

## Discussion

Accumulating evidence suggests the potential of tDCS to treat eating and weight disorders, while bariatric surgery outcomes leave room for improvement. The present study firstly investigates the combination of tDCS, a personalized food-specific inhibitory control training and a structured psychosocial intervention after bariatric surgery based on a precursor randomized-controlled pilot study in BED [27, 28]. The current pilot study will provide valid information on the potential of neuromodulatory treatments to enhance bariatric surgery outcomes and inform randomized-controlled trials in larger samples regarding effect sizes, acceptability, feasibility and safety.

Strengths of the study design are the incorporation of brain-based (i.e., tDCS), behavioral (i.e., inhibitory control training) and psychological (i.e., structured intervention) aspects, suited to disentangle singular from additive effects of neuromodulatory versus psychosocial treatments. Physical and mental state is comprehensively assessed through self-report, interview and hormonal markers. Meta-analytic notions on cumulative effects of tDCS on food craving and consumption [59] resulted in the number of six sessions. The double-blind study design was tested thoroughly in BED [27, 28], suited to different effects of verum from sham tDCS. We consider a 3-month follow-up as sufficient to determine the maintenance of changes after termination of the intervention.

Challenges include an increased risk for drop-out as bariatric surgery candidates may not maintain their motivation after surgery against the common ongoing negotiation and ambivalence after bariatric surgery [60]. The cooperation with the University Clinic for General, Visceral, Vascular and Transplant Surgery throughout the whole procedure aims to enhance patients’ confidence, positive treatment expectations and compliance. Importantly, the drop-out rate of the present trial will be compared to the precursor study in BED [27, 28], which demonstrated high acceptability of the intervention. Obesity and BED share overlapping neurobiological features, giving rise to the transfer of the study design tailored to a sample with BED [27, 28] to the present sample of patients after bariatric surgery. Essentially, prefrontal dysfunctionalities are assumed to be pronounced in BED compared to patients with obesity [22, 61, 62]. Results will clarify whether the current intervention dedicated towards reward and executive functioning will thus prove more acceptable and effective [63] in BED [27, 28] compared to the current sample of patients after bariatric surgery. Notwithstanding these considerations, possibly higher odds to drop out of patients with obesity compared to patients with BED bear a risk to undermine the findings of the present trial (i.e., the estimation of the effect of the intervention). The behavioral assessments of the antisaccade task at t1 and t2 were deleted in favor of a concise patient-friendly assessment, limiting longer-term conclusions to self-report and interview-based outcomes. While patients are asked to attend the lab in a fasted state, possible violations of this requirements are only assessed in a dichotomous manner, but not quantified, possibly introducing variability. Lastly, patients are unfamiliar with tDCS, which may lead to concerns about safety and adverse effects. However, a thorough psychoeducation was conceptualized to allay these doubts, based on the demonstrated effect of tDCS plus psychoeducation compared to the respective mono-interventions in smokers [64].

In summary, the current trial provides a first step to bridge unmet treatment needs of patients after bariatric surgery, who repeatedly ask for clinical appointments after surgery. The standard treatment includes few postoperative appointments, notwithstanding the recommendation for postoperative interventions in clinical guidelines [5]. Results will provide the basis for cost-benefit analyses, comparing the effects of the relatively low-cost state-of-the-art treatments with the novel treatment adjuncts presented here and, potentially, with emerging successful potent but expensive pharmacotherapies [65].

## Electronic supplementary material

Below is the link to the electronic supplementary material.

Supplementary Material 1

## Data Availability

No datasets were generated or analysed during the current study.

## Abbreviations

BDI: Beck Depression Inventory
BIS-15: Short version of the Barratt Impulsiveness Scale
DLPFC: Dorsolateral prefrontal cortex
EDE-BSV: Eating Disorder Examination Interview – Bariatric Surgery Version
EDE-Q: Eating Disorder Examination Questionnaire
FAAQ: Food Craving Acceptance and Action Questionnaire
FCQ-S: Food Cravings Questionnaire-State
FCQ-T: Food Cravings Questionnaire-Trait-reduced
FEV: Three Factor Eating Questionnaire
GLP-1: Glucagon like peptide-1
Mini-DIPS: Diagnostisches Kurz-Interview bei psychischen Störungen
PHQ-D: Patient Health Questionnaire
PYY: Peptide YY
QOLOS: Quality of Life for Obesity Surgery
t0: Baseline after the sleeve gastrectomy
t1: 4 weeks after the tDCS intervention
t2: 3 months after the tDCS intervention
tDCS: Transcranial direct current stimulation
UPPS: Impulsive Behavior-Scale
WHO-5: Well-being index

## Footnotes

1 State impulsivity: Individuals with obesity might experience heightened state impulsivity in situations in which they are exposed to food cues, are under stress, or experience negative emotions. This can lead to impulsive eating behaviors, such as binge eating or overeating, even when they are not physically hungry.

2 Food-specific impulsivity: Individuals with obesity might exhibit a stronger drive to consume or find it more difficult to resists tempting foods. This can be due to altered neural responses in the brain’s reward system and PFC, making it harder to exert self-control over eating behavior.

## Declarations

### Author’s information

SAR worked for several years as a clinical psychologist in a Day Clinic for Obesity Treatment, which provides a conservative multimodal weight loss program and a structured psychosocial program after bariatric surgery and wrote her PhD thesis on neural correlates and the efficacy of neurofeedback for BED and obesity.
