# Supplementary material for: Enhancing the outcomes of bariatric surgery with inhibitory control training, electrical brain stimulation and psychosocial aftercare: a pilot study protocol
Source: J Eat Disord. 2024 Dec 9;12:202. doi: 10.1186/s40337-024-01160-3 (PMC11626763; doi:10.1186/s40337-024-01160-3)
Supplement: Supplementary file 1 — Supplementary Material 1 [file 40337_2024_1160_MOESM1_ESM.docx]

**Online Supplementary Material for:**

Rösch, S., Wünsche, L., Reinstaller, T., Thiele, C., Zähle, T., Schag, K., Giel, K. E., Plewnia, C., Steiner, J., Junne, F.

# Supplementary Methods

## The Translation of the FAAQ

All items of the FAAQ [1], including the anchor scales, were translated into German by the native-speaking German first author who has specific expertise in the field of disordered eating (Translation 1, Table 1). The lower anchor of the scale (1 = *very seldom true*) was translated to 1 = *sehr selten*, the upper anchor of the scale (6 = *always true*) was translated to 6 = *immer*. This initial translation was reviewed by 5 independent members of the research team otherwise unrelated to the study, who rated the correspondence between the original English items and the anchor scales and the translated German items on a 4-point Likert scale ranging from 1 = no correspondence (1 =*keine Übereinstimmung*), 2 = low level of analogy (2 =*geringe sinngemäße Übereinstimmung*), 3 = high level of analogy (3 = *hohe sinngemäße Übereinstimmung*), to 4 = high correspondence (4 = *hohe Übereinstimmung*). Ratings are depicted in Table 1; the mean rating of the initial translation was *M* = 3.86 (*SD* = 0.35), the correspondence of the upper and the lower anchor scales was *M* = 3.5 (*SD* = 0.5). All members of the research team were also invited to suggest alternative translations. Based on the ratings and the provided alternative translations, 5 of the 10 translations were reviewed and adapted by the first author and a trained research assistant (MSc level). The remaining items were retained in their initial translation. This resulted in a preliminary German version that was approved for the purpose of the study (Table 1; German Translation 2). The same 5 members of the research team were asked to rate the final translation, yielding a correspondence of *M* = 3.86 (*SD* = 0.35).

**Table 1.**

Translation of the FAAQ

| Item | English Version | German Translation 1 | *M* (*SD*)^a^ | German Translation 2^b^ | *M* (*SD*) ^a^ |
| --- | --- | --- | --- | --- | --- |
| 1 | *I continue to eat a healthy diet, even when I have the desire to overeat or make poor eating choices* | *Ich ernähre mich weiterhin gesund, selbst wenn ich das Verlangen habe, mich zu überessen oder schlecht zu ernähren.* | 3.8 (0.4) |  |  |
| 2 | It's OK to experience cravings and urges to overeat, because I don't have to listen to them. | Es ist okay, Gelüste nach übermäßigem Essen zu verspüren, denn ich muss diesen nicht nachgehen. | 3.8 (0.4) | Es ist okay, Gelüste und den Drang nach übermäßigem Essen zu verspüren, denn ich muss diesen nicht nachgehen. | 4 (0) |
| 3 | *It's not necessary for me to control my food urges in order to control my eating.* | *Ich muss meinen Drang zu essen nicht kontrollieren, um mein Essverhalten zu kontrollieren.* | 3.8 (0.4) | *Es ist nicht notwendig, dass ich meine Essensgelüste kontrolliere, um mein Essverhalten zu kontrollieren.* | 3.8 (0.4) |
| 4 | I need to concentrate on getting rid of my urges to eat unhealthily. | Ich muss mich darauf konzentrieren, meine Gelüste, ungesund zu essen, loszuwerden. | 4 (0) |  |  |
| 5 | I don't have to overeat, even when I feel like I want to overeat. | Ich muss mich nicht überessen, selbst wenn ich das Gefühl habe, dass ich mich gern überessen würde. | 4 (0) |  |  |
| 6 | *Controlling my urges to eat unhealthily is just as important as controlling my eating.* | *Es ist genauso wichtig, meine Gelüste, ungesund zu essen zu kontrollieren wie mein Essverhalten selbst zu kontrollieren.* | 3.8 (0.4) |  |  |
| 7 | My thoughts and feelings about food must change before I can make changes in my eating. | Meine Gedanken und Gefühle über das Essen müssen sich zuerst ändern, bevor ich mein Essverhalten ändern kann. | 3.8 (0.4) | Meine Gedanken und Gefühle in Bezug auf das Essen müssen sich ändern, bevor ich mein Essverhalten ändern kann. | 4 (0) |
| 8 | Despite my cravings for unhealthy foods, I continue to eat healthily. | Trotz meiner Gelüste nach ungesundem Essen ernähre ich mich weiterhin gesund. | 4 (0) |  |  |
| 9 | Before I can make any important dietary changes, I have to get some control over my food urges. | Bevor ich meine Ernährung umstellen kann, muss ich Kontrolle über meine Essensgelüste bekommen. | 3.8 (0.4) | Bevor ich eine wichtige Ernährungsumstellung vornehmen kann, muss ich Kontrolle über meine Essensgelüste bekommen. | 3.2 (0.4) |
| 10 | Even if I have the desire to eat something unhealthy, I can still eat healthily. | Auch wenn ich Lust habe, etwas Ungesundes zu essen, kann ich trotzdem etwas Gesundes essen. | 3.8 (0.4) | Auch wenn ich das Verlangen habe, etwas Ungesundes zu essen, kann ich trotzdem gesund essen. | 4 (0) |

^a^ based on the ratings of 5 members of the research team regarding the correspondence of the translation with the original version. ^b^ based on the suggested alternative translations of the members of the research team, the first author and a trained research assistant (MSc level) discussed about appropriate translations and yielded a consensus.

# Supplementary Discussion

Ideally, future studies should follow the guidelines of the World Health Organization [2] for the translation and adaptation of instruments, proposing a forward-backward translation rationale. Consequently, a native English-speaker needs to translate the current approved preliminary version into English. Lastly, the back-translated version needs to be reviewed and compared with the English original version by the study team and the original author. This approach would improve the validity and ensures to retain the original meaning of the items.

# Literature

1. Juarascio A, Forman E, Timko CA, Butryn M, Goodwin C. The development and validation of the food craving acceptance and action questionnaire (FAAQ). *Eat Behav*. 2011;12(3):182-187. doi:10.1016/j.eatbeh.2011.04.008
2. World Health Organization (2016). Process of translation and adaptation of instruments. Geneva. Available from: http://www.who.int/substance_abuse/research_tools/translation/en/, accessed 17 Mai 2024
